# PEN-13: A New Generic 13-Item Questionnaire for Measuring Patient Enablement (German Version)

**DOI:** 10.3390/ijerph16234867

**Published:** 2019-12-03

**Authors:** Achim Siegel, Anna T. Ehmann, Ingo Meyer, Oliver Gröne, Wilhelm Niebling, Peter Martus, Monika A. Rieger

**Affiliations:** 1 Institute of Occupational and Social Medicine and Health Services Research, University Hospital Tübingen, Wilhelmstraße 27, 72074 Tübingen, Germany; anna.ehmann@med.uni-tuebingen.de (A.T.E.); monika.rieger@med.uni-tuebingen.de (M.A.R.); 2 PMV Forschungsgruppe, University of Cologne, Herderstraße 52, 50391 Cologne, Germany; ingo.meyer@uk-koeln.de; 3 OptiMedis AG, Burchardstraße 17, 20095 Hamburg, Germany; o.groene@optimedis.de; 4 London School of Hygiene and Tropical Medicine, University of London, London WC1E 7HT, UK; 5 Division of General Practice, University Medical Center Freiburg, 79910 Freiburg, Germany; wilhelm.niebling@googlemail.com; 6 Institute for Clinical Epidemiology and Applied Biometry, University Hospital Tübingen, Silcherstr. 5, 72076 Tübingen, Germany; peter.martus@med.uni-tuebingen.de

**Keywords:** standardized questionnaire, validation, patient enablement, patient empowerment, patient activation

## Abstract

*Background*: The purpose of our study was to develop and psychometrically test a German-language survey instrument that measures patient enablement generically and in greater detail than previous instruments. *Methods*: A multidisciplinary team developed 13 items to capture individual aspects of patient enablement (PEN-13). A pre-test with 26 subjects was followed by a random sample survey of *N* = 1168 subjects. An exploratory factor analysis was conducted in a random split-half sample of the data to explore PEN-13’s factor structure; a confirmatory factor analysis was conducted in the validation sample. The internal consistency of the factors was evaluated using Cronbach’s alpha, PEN-13’s construct validity was checked by means of additional hypothesis testing. *Results*: The two factors self-management and patient-practitioner interaction, detected in the exploratory analysis, were confirmed with a few modifications in the confirmatory factor analysis, with the comparative fit index (CFI) amounting to 0.903. The Cronbach’s alpha values of those two factors amounted to α = 0.90 and α = 0.82, respectively. The correlations of the PEN-13 score with the ’general self-efficacy’ and ’health literacy’ (HLS-EU-Q16) scores further confirmed its construct validity; the respective correlation coefficients amounted to 0.57 and 0.60. *Conclusion*: The German version of the survey instrument Patient Enablement Scale—13 items (PEN-13) shows acceptable psychometric properties. *Practical implications*: PEN-13 seems particularly suitable for health services research purposes. We recommend checking the results in another sample as well as evaluating its responsiveness to enablement-enhancing interventions.

## 1. Introduction

Patient enablement refers, broadly speaking, to the process or the result of enabling patients to assess and manage their health conditions more competently, both as individuals and within the practitioner-patient relationship. Patient enablement is a core element of patient empowerment and patient activation [1], and as such is an important goal of contemporary health policy. All three concepts, i.e., patient enablement, empowerment, and activation, can be (and have been) defined either as a process or as an emergent state. As e.g., Fumagalli et al. summarized several conceptualizations [2,3,4,5], patient enablement may be defined as the process of enabling patients by “(1) providing appropriate knowledge, skill and abilities to understand their conditions and make decisions; and (2) developing appropriate contexts that allow patients to learn such knowledge, skill and abilities.” [1]. Patient enablement may also be defined as an emergent state reflecting “the gained measure in which patients understand their health conditions and feel able to cope with them” [1,6]. The same holds for the terms ‘patient empowerment’ and ‘patient activation’: Both can be conceptualized as either a process or an emergent state [1].

The relevance of patient enablement in the health services context has been shown in numerous studies which have been analyzed and summarized by Hudon et al. in their concept analysis [7] and in an integrative review by Frost et al. [8]. According to Hudon et al., the consequences of patient enablement include, e.g., patient satisfaction, a feeling of self-efficacy, the development of certain skills, improvement in patients’ health condition and quality of life as well as in their participation in care, but also higher job satisfaction and self-confidence in health professionals [7]. Other important consequences are a reduced dependency of patients on health services, better consultations [8], and a reduced preference in patients for seeing a different doctor [9].

But can we conceive ‘patient enablement’ on the one hand and ‘patient empowerment’ and ‘patient activation’ on the other hand as distinct concepts? One should keep in mind that existing conceptualizations of each of these terms are heterogeneous, in particular with regard to ‘patient empowerment’ [10,11]. However, it is broadly possible to detect some dominating tendencies in the usage of those concepts [1,11]. In past research, ‘patient enablement’ and ‘patient empowerment’ were often used as synonyms [1]. Referring to the arguments of Fumagalli et al. [1] and Castro et al. [11] we can distinguish the two terms by referring to the elements of self-determination, power, motivation, and engagement: Patients are ‘enabled’ when they are *able* to engage in self-care or to take part in shared decision-making, but not necessarily have the power and/or motivation to do so (because, e.g., they do not have a sufficiently strong desire for self-determination). In comparison, patients are ’empowered’ when they are not only able to engage in self-care and shared decision-making but also have a sufficiently strong desire for self-determination and the power—and thus the motivation—to do so. Lacking self-determination and power as defining attributes, the term ‘enabled patient’ has a narrower connotation (intension) but a larger conceptual scope (extension) than the term ‘empowered patient’.

**Figure 1:**
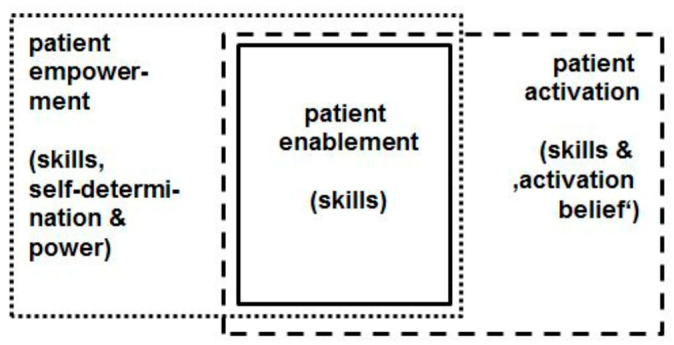
Connotation map of ‘patient enablement’, ‘patient empowerment’, and ‘patient activation’.

**Figure 2:**
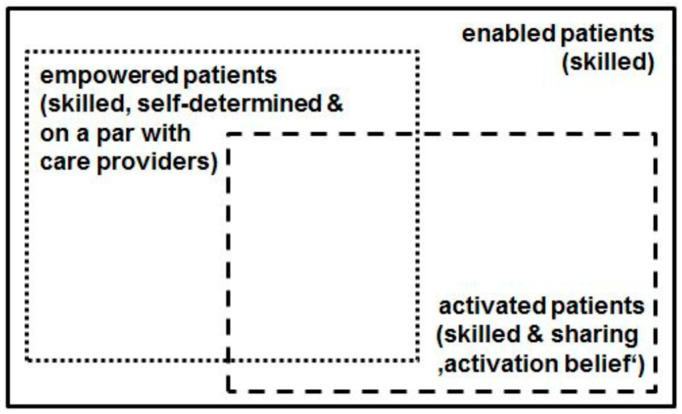
Conceptual scope map (scheme) of ‘enabled patient’, ‘empowered patient’, and ‘activated patient’.

The comparably recent concept ‘patient activation’, put forward in the preceding decade by Hibbard et al. [12,13,14,15], comes likewise very close to ‘patient enablement’: According to Hibbard’s conceptualization, activated patients “believe patients have important roles to play in self-managing care, collaborating with providers, and maintaining their health. They know how to manage their condition and maintain functioning and prevent health declines; and they have the skills and behavioral repertoire to manage their condition, collaborate with their health providers, maintain their health functioning, and access appropriate and high-quality care.” [15]. Hibbard’s ‘Patient Activation Measure’ shows, even in its common short form (PAM-13) [12], that the ‘belief component’ is crucial: Activated patients believe that they have a crucial role in managing their own healthcare; moreover they are confident that they are able to apply their knowledge and skills, and therefore are motivated to do so. The importance of the belief component is reflected e.g., in the first two items of the PAM-13 questionnaire: (i) “When all is said and done, I am the person who is responsible for managing my health condition” and (ii) “Taking an active role in my own healthcare is the most important factor in determining my health and ability to function” [12]. Unlike ‘patient enablement’, the term ‘patient activation’ conceives a specific belief as a key attribute—in addition to knowledge and skills. Thus we may conclude that the connotation of the ‘enabled patient’ is also narrower than the connotation of the ‘activated patient’, implying its scope is larger than the conceptual scope of the ‘activated patient’. Figure 1 and Figure 2 illustrate our argument: Figure 1 provides a connotation map of the terms ‘patient enablement’, ‘patient empowerment’, and ‘patient activation’; Figure 2 shows a scheme of the conceptual scopes of the terms ‘enabled patient’, ‘empowered patient’, and ‘activated patient’.

The reason for the present study was our experience with an own trend study, in which we surveyed patient-related outcomes in an integrated healthcare system in Germany [16]. Here, we originally wanted to include a measure of either patient activation or patient enablement as these were strategic aims of the integrated healthcare system management. Furthermore, we sought a generic instrument applicable across health indications and valid for persons with chronic or acute conditions. So we took a closer look at Hibbard’s PAM-13 [12] and its German version [13] and at Howie’s seminal ‘Patient Enablement Instrument’ (PEI) [2,17]. We decided not to use PAM-13 because of three reasons: First, some items of PAM-13 seem to match patients in chronic conditions very well [12,15] whereas they do not so well apply to patients with non-chronic conditions (e.g., item “I know what each of my prescribed medications do”). Second, the PAM-13 questionnaire is provided by a commercial company and therefore is not free to use [14]. And third, after a closer look at the ‘confidence’ and ‘belief’ items we thought it might be more reasonable to focus on (self-perceived) knowledge, skills, and abilities, and to leave the ‘belief’ component aside: e.g., the PAM-13 item “Taking an active role in my own healthcare is the most important factor in determining my health and ability to function” may be perceived as overstated or overgeneralized, reflecting rather an ideology than an empirically verifiable generalization. Howie’s PEI, on the other hand, is a well-established instrument measuring patient enablement with six items. Derived from qualitative research with patients on what mattered most to them in terms of consultation outcomes [17], the PEI has a high internal consistency (Cronbach’s alpha = 0.93 [2]). Meanwhile, the PEI has been translated into many other languages and tested in different countries where its high internal consistency has been confirmed [9,18,19,20,21,22,23], with the respective Cronbach’s alpha values ranging from 0.84 (Chinese version [22]) to 0.93 (English original version [2] and French version [19]). Although test-retest reliability has sometimes been found only moderate [19,21], the different PEI versions have good psychometric properties. A disadvantage of the PEI, however, seems to be that it requires a prior intervention (e.g., a physician-patient consultation) as the questionnaire items directly measure patients’ perceived change in skills etc. in response to a given intervention. The PEI, then, is a *direct* measure of perceived change in enablement and does not measure a given level of enablement, as Enthoven et al. clearly stated [18]. This might lead to findings that seem paradoxical when the PEI is taken as an indicator of patient enablement: Thus, e.g., patients with less experience and knowledge of their disease might be more likely to improve in terms of the PEI score than patients who have experienced problems for a longer time, have tried several (self-) treatment options and might be real ‘experts’ of their own disease [18]. However, because the PEI is a direct measure of change of enablement (and not of the level of enablement), the first patient in our example might appear to be ‘more enabled’ than the second one—on the grounds of the PEI. For the same reason, the PEI cannot serve well as a simple survey instrument in a population which has not been subjected to a particular intervention. Furthermore, since PEI comprises only six items, it might be a too ‘general’ or ‘global’ measure for some specific ends. For example, none of the six PEI items explicitly refers to a patient’s ability to appropriately interact with health professionals—an aspect which might be considered an important enablement component (e.g., [5,7]). For these reasons we decided to develop and validate a new generic measure of patient enablement which is more detailed than PEI and not bound to a previous intervention.

In addition, to support widespread quality improvement efforts and monitoring of patient enablement in the population [24], the new instrument should be freely available. As we planned to use this new measure first in a German healthcare setting, we designed it in German language. But from our own experiences in European research projects we conclude that there is a demand to translate and validate the instrument also in other languages.

## 2. Materials and Methods

### 2.1. Item Development

The analysis of the concepts ‘patient enablement’ [2,17], ‘patient activation’ [12,13,14,15] and ‘general self-efficacy’ [25] served as the initial basis for the development of the items of our questionnaire.

Thereafter, a multidisciplinary team (consisting of one general practitioner, one specialist in occupational medicine, one sociologist and two public health researchers) formulated items that were to cover individual aspects of patient enablement independently of a particular medical indication or intervention. The generic approach should make it possible to compare the extent of patient enablement in patients with different chronic diseases, but also in people with acute medical conditions. Therefore, the questionnaire should not only include items that address knowledge and competence in relation to managing one’s own diseases but also items that address knowledge about possibilities of health promotion and prevention as well as general aspects of effective communication with doctors or other health professionals. Thus, e.g., we included items that read “I know how I can promote my health”, “It is easy for me to practice health-promoting behavior in everyday life (e.g., nutrition, exercise)” and “It is easy for me to ask questions or express my wishes during a medical consultation”. To check the content validity and comprehensibility of the items, we conducted a pre-test with 26 test persons; these were recruited from insureds of a statutory health insurer who were enrolled in the integrated healthcare system ‘Gesundes Kinzigtal’ [26,27] and from employees of the management company Gesundes Kinzigtal GmbH. According to their feedback we adapted the wording of the items for the sake of greater clarity and better understanding. This resulted in a version with 13 items. (At the same time, a cultural adaptation of the German instrument in English was carried out; the adapted provisional English version is presented in Table 3). The answers to the 13 items can be given on a five-point Likert scale (strongly disagree; disagree; neither/nor; agree; strongly agree). In the following, the resulting measurement scale is named PEN-13 (short for ‘Patient Enablement Scale’—13 items). Comprising 13 items, PEN-13 total score values can range from 13 (minimum enablement) to 65 points (maximum enablement).

### 2.2. Data Collection and Study Population

The piloted items were part of a more comprehensive survey in a trend study [16] with registered ‘Gesundes Kinzigtal’ members. The study had been positively reviewed by the Ethics Committee of the University of Freiburg (Az. 294/12_140826). On the basis of 75 questionnaire items, the participants were asked about their satisfaction with their trusted physician and the integrated care system. Validated survey instruments in the questionnaire included the EQ-5D (3-level version) and the respective Visual Analogue Scale (EQ-VAS) to assess the subjects’ view on their own health [28].

In summer 2017, 3218 registered members of the integrated care system were randomly selected and asked to participate in the survey. By returning the questionnaire, they gave informed consent to participate in the study. The absolute response of the survey was 36.7%; 1168 questionnaires (36.3%) could be included in the analysis. From the 1168 survey participants with evaluable questionnaires, 180 had completed their questionnaires with two additional scales which were used for construct validation. They are described in greater detail in Section 2.3.

### 2.3. Scales for Construct Validation by Hypotheses Testing

To evaluate the construct validity by hypotheses testing [29,30], we used the following two instruments: The Generalized Self-Efficacy Scale (GSE) [25,31] is an internationally standardized measuring instrument and consists of ten items that capture the general self-efficacy unidimensionally [25,31]. When comparing GSE scores across 25 countries, the internal consistency values (Cronbach’s alpha) ranged between 0.75 and 0.91, and the mean Cronbach’s alpha for Germany was 0.81 [32]. Respondents’ health literacy was measured using the instrument HLS-EU-Q16 (European Health Literacy Survey Questionnaire, short version) [33], with its 16 items representing four dimensions. With a Cronbach’s alpha of 0.90, the HLS-EU-Q16 showed a high internal consistency in a German study [34].

### 2.4. Statistical Analysis

This study is based on classical test theory. The Consensus-based Standards for the Selection of Health Measurement Instruments (COSMIN) checklist [29,30,35] was used as a guideline for reporting [36]. The total sample was randomly divided into two equal subsamples, a calibration sample (*n* = 584) and a validation sample (*n* = 584). The socio-demographic characteristics of the total sample and the two subsamples were evaluated descriptively. The comparability of the two subsamples was tested with t-tests, Mann-Whitney-U-tests or Chi^2^-tests.

To identify items that have a low correlation (<0.5) with the PEN-13 scale as a whole, we first looked at the matrix of item-total correlations. In the calibration sample (*n* = 584), we performed an exploratory factor analysis with Varimax rotation to obtain a single structure. This method is used to reduce data, i.e., to find a smaller number of underlying factors in a large number of items. The Kaiser-Meyer-Olkin criterion was used to assess the suitability of the data. A value equal to or higher than 0.6 indicates suitability [37]. The factors were then selected according to the Kaiser criterion (eigenvalue > 1), interpreted and named with regard to the corresponding items.

### 2.5. Reliability

As one aspect of reliability [29,30], the internal consistency of the individual factors was assessed in the validation sample using the Cronbach’s alpha coefficient. A value of α ≥ 0.7 per factor is considered to be good [38].

### 2.6. Construct Validity

#### 2.6.1. Structural Validity

Structural validity [29,30,35] was examined by carrying out a confirmatory factor analysis. This validation was performed on the basis of the data of the second subsample (*n* = 584) using confirmatory factor analysis (CFA) with maximum likelihood estimation. The goodness of fit can be assessed on the basis of various indices. The quality of the model fit—derived from the exploratory factor analysis (EFA)—was evaluated on the basis of the comparative fit index (CFI), Tucker Lewis Index (TLI) and Root Mean Square Error of Approximation (RMSEA) with its 90% confidence interval. We considered the model fit acceptable if CFI > 0.90 [39,40]. TLI and RMSEA values were considered as secondary (additional) fit measures. Usually, TLI values close to 1.0 indicate good fit and RMSEA values < 0.1 acceptable fit. Note that no generally accepted cutoffs for fit indices can be accepted, as Bollen writes in his standard textbook [41]: Any value (of an incremental fit index) will be controversial. Modifications to improve model fit were based on modification indices from Amos output, showing which adjustments improve model fit the most, as well as on theoretical considerations. We only allowed additional correlations if the relationship between the items was theoretically reasonable and the model fit improved. To have the modification indices calculated, the missing values were replaced by the sample mean value in Amos.

#### 2.6.2. Hypotheses Testing

The construct validity was checked with correlation analyses [29,30,35] focusing on convergent validity. This is the extent of agreement with test results of similar characteristics [42]. Correlations of 0.1 are considered low, 0.3 medium and above 0.5 high [43]. A total PEN-13 score was calculated if at least 10 of the 13 items had been answered. In this case, missing values were substituted by the mean of the respondent’s valid items. For the evaluation of construct validity by hypothesis testing, the following four hypotheses were stated.

**Hypotheses 1** **(H1).** *There is a high positive correlation between the PEN-13 score and the General Self-Efficacy score [25]. This hypothesis is based on the similar content of the items; notwithstanding, general self-efficacy is an even more generic concept than patient enablement [44].*

**Hypotheses 2** **(H2).** *There is a moderate to high positive correlation between the PEN-13 score and the health literacy score HLS-EU-Q16 [33,34]. Whereas Smith et al. concluded that health literacy and patient activation are only weakly correlated with each other, and also make independent contributions to health [45], we hypothesize a stronger correlation between patient enablement and general health literacy, since both constructs conceptualize similar aspects in parts.*

**Hypotheses 3** **(H3).** *There is a moderate positive correlation between the PEN-13 score and the assessment of personal health status using EQ-VAS. As a systematic review concluded a relationship between low health literacy and poorer health status [46], we similarly suppose an effect of patient enablement on the health status (and vice versa).*

**Hypotheses 4** **(H4).** *There is a low positive correlation between the PEN-13 score and the highest school-leaving certificate of the respondents.*

Pearson’s correlation coefficient was used to test the first three hypotheses, whereas Spearman’s rank correlation coefficient was used to test hypothesis 4. For the test of hypotheses 1 and 2 only the questionnaires of the subsample (*n* = 180) with the two additional scales could be used. All analyses were performed with SPSS version 25 (IBM Analytics, IBM Corporation, Armonk, NY, USA)) and Amos 25 (IBM Analytics, Amos Development Corporation, Wexford, PA, USA).

## 3. Results

### 3.1. Response and Socio-Demographic Characteristics

The response rate of the whole survey was 36.3%. The socio-demographic data of the total sample and the two subsamples are presented in Table 1. More than half of the respondents were women (56.7%). The average age of the survey participants was 62 years (SD = 15.9), the median age 64 years. Somewhat more than half of the respondents stated that they suffered from one or more chronic diseases. Table 2 shows the description of the other health-related scales.

The comparability of the two subsamples was verified: The socio-demographic characteristics age (T = −1.450; *p* = 0.147), gender (Chi^2^ = 0.056; *p* = 0.859), chronic illness (Z = −0.102; *p* = 0.919), highest school-leaving qualification (Z = −0.189; *p* = 0.850), current employment (Chi^2^ = 0.335; *p* = 0.563), health-related quality of life (EQ-5D Index: T = 0.658; *p* = 0.511) and personal health (EQ-VAS: T = 0.673; *p* = 0.276) did not differ significantly in the two subsamples.

### 3.2. Exploratory Analysis

All 13 items correlated with the overall PEN-13 scale with *r* ≥ 0.5. The suitability of the data for an EFA was proven with Kaiser-Meyer-Olkin criterion = 0.925 (Bartlett Test *p* ≤ 0.001). After rotation, two factors could be determined. The results of the EFA are provided in Table S1. The two factors can be termed ‘self-management’ (items 1–7 and items 11–13) and ‘patient-practitioner interaction’ (items 8–10). Factor 1 explains 36.4% and factor 2 explains 25.4% of the variance. The wording and statistical measures of the 13 items as well as the factor loadings are shown in Table 3. Table 4 shows the inter-item-correlation matrix of the total sample and Table 5 the corrected item-total correlations in the total sample.

A valid PEN-13 score could be calculated for 1103 respondents (94.4% from *N* = 1168). The mean value of the total score of all respondents who had completed at least 10 items was M = 52.12 (SD = 8.55). As to the two factors, the mean value of the items of factor 1 with 3.94 (*n* = 972; SD = 0.67) is significantly lower (*p* < 0.001) than the mean value of the items of factor 2 with 4.18 (*n* = 1103; SD = 0.81). One survey participant (0.1%) achieved the lowest possible score reaching 13 points, while 77 survey participants (6.6%) reached the highest possible score (65 points).

With the aid of a t-test and Chi^2^-test, participants with or without valid PEN-13 score were compared with respect to the following characteristics: age, chronic disease, gender, health-related quality of life and personal health status. Participants with a valid PEN-13 score were significantly younger than participants with no valid PEN-13 score (T = 4.919; *p* < 0.001); participants with a valid PEN-13 score indicated more often a chronic disease than participants with no valid PEN-13 score (Chi^2^ = 7.175; *p* = 0.011; Cramer’s V = 0.080); the two groups with or without valid PEN-13 score, respectively, did not differ significantly regarding gender, health-related quality of life (EQ-5D Index) and personal health (EQ-VAS).

### 3.3. Internal Consistency

In the validation sample, the internal consistency was α = 0.90 for factor 1 (*self-management*, 10 items) and 0.82 for factor 2 (*patient-practitioner interaction*, three items). The internal consistency for all 13 items—treated as one factor—was α = 0.91.

### 3.4. Confirmatory Analysis

**Figure 3:**
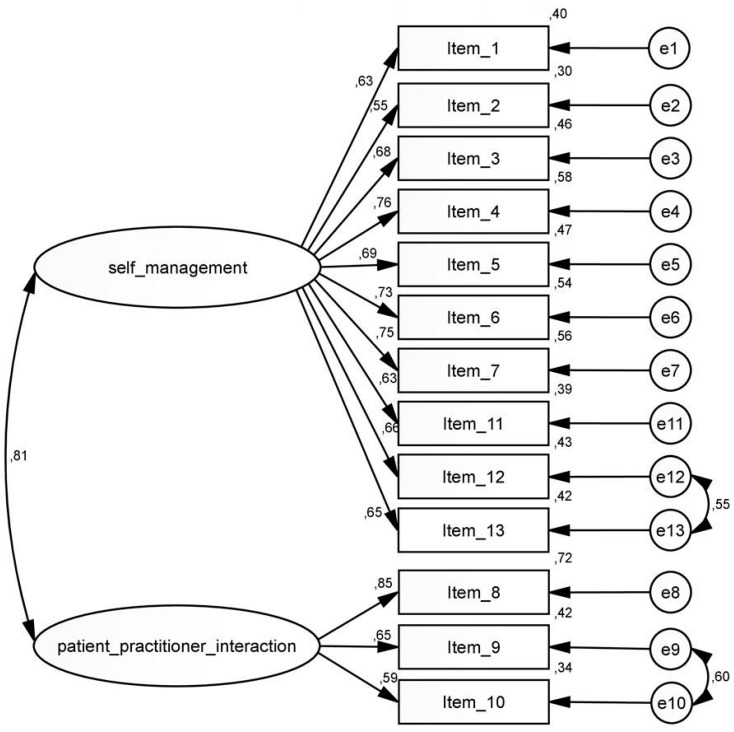
Path model for the CFA of the German PEN-13 and the corresponding path coefficients. Explication of Figure 3: The values between the factor and the item are the corresponding path coefficients; the values in the right column (0.40; 0.30; 0.46; …) are the path coefficients with the error terms.

In the CFA, the two-factor model with 13 items (see Figure 3) showed a model fit of CFI = 0.821 and was better than the one-factor model (CFI = 0.765). With two adjustments to the model, allowing two correlations within the same factor, i.e., between the error terms of items 12 and 13 (factor 1) and items 9 and 10 (factor 2), the model fit of the two-factor model improved to CFI = 0.903. 

As to the one-factor model, similar adjustments also led to a higher model fit which, in the end, was still a little poorer compared to the model fit of the two-factor model with the two adjustments (see Table 6). The interrelationship between the two selected items is in each case plausible: The two items of the first factor (12 and 13) refer to a general way of coping in life; the two items of the second factor (9 and 10) are about being able to easily address concerns in a medical consultation (for the wording of the items see Table 3).

To assess the practical impact of the model adjustments on the basis of modification indices, the estimated factor scores for the model with the two adjustments and the total PEN-13 scores for the two factors were correlated. The correlations with the score for factor 1 and the factor score with estimated weights of factor 1 is *r* = 0.994 and with the score for factor 2 and the estimated weights of factor 2 *r* = 0.913, respectively. Figure 3 shows the CFA model with the specific modifications for the German version of the scale with the corresponding path coefficients.

### 3.5. Hypotheses Testing

The hypotheses for convergent validity with the PEN-13 score in the validation group could be confirmed.

**Hypotheses 1** **(H1).** *The PEN-13 and GSE scores correlated highly with each other (r = 0.57; p < 0.001; n = 158; 95% CI: 0.46–0.67).*

**Hypotheses 2** **(H2).** *There was also a high correlation between the PEN-13 score and the health literacy score HLS-EU-Q16 (r = 0.60; p < 0.001; n = 125; 95% CI: 0.47–0.70).*

**Hypotheses 3** **(H3).** *A moderate correlation resulted between the PEN-13 score and the personal state of health (EQ-VAS) on the survey day (r = 0.41; p < 0.001; n = 500; 95% CI: 0.33–0.48).*

**Hypotheses 4** **(H4).** *There was a low correlation between the PEN-13 score and the level of education (r_s_ = 0.15; p < 0.001; n = 547; 95% CI: 0.07–0.24).*

## 4. Discussion

In our study we developed and validated a measure of patient enablement to assess it in a comprehensive manner; the use of this measure is independent of a previous intervention and conceptually distinct from both patient activation and patient empowerment.

Our results show that the Patient Enablement Scale-13 items (PEN-13) is suitable for providing reliable and valid results for the measurement of patient enablement in a sample with different types and severity of medical conditions.

The results of the exploratory factor analysis show that the PEN-13 is characterized by two factors. The internal consistency of both factors achieved good to excellent values; its level is comparable to that of the PEI (see the ‘Introduction’ section) and the PAM-13 [12,13,14,47,48,49,50,51,52,53]. The confirmatory factor analysis verified the two factors self-management and patient-practitioner interaction. Adjustments to this two-factor model additionally improved the values for model fit. Due to the high correlations between the original and re-weighted factor scores we assume that the adjustments had no relevant impact on the score. Since no practical effects on the scores are expected, these adjustments were permitted in favor of a slightly improved model fit. The application of these adjustments in other samples must be verified. Further validation studies will be helpful to confirm or reject the two additional correlations.

Due to the high correlation between the two factors (*r* = 0.81), it could also be justified to group the items into one factor. In this case, however, a poorer model fit resulted. In addition, the aim of the study was to find out more about the principle—and the ‘inner structure’—of the ‘enabled patient’. The comparison with the one-factor model showed then that the conceptualization of two underlying factors should be preferred to the one-factor conceptualization. As the items are not bound to a specific treatment situation or health indication, the PEN-13 matches persons with acute or chronic illness. Nevertheless, it should be noted that participants who stated that they did not suffer from any chronic disease completed the scale less often than participants who indicated a chronic disease (see Table 3 for missing values in item 5 and the last paragraph in Section 3.2.).

For the construct validation with correlation analyses, positive correlations with the Generalized Self-efficacy Scale (GSE) and the 16-item version of the European Health Literacy Survey Questionnaire (HLS-EU-Q16) were expected. Due to the similarity of the item formulations of the GSE, a high correlation was assumed and confirmed with *r* = 0.57. A moderate to high correlation was expected between the PEN-13 score and the health literacy score; the result in our study (*r* = 0.60, *p* < 0.001) confirmed these expectations. Moreover, health literacy seems to be closely linked to patient empowerment [54]. Finally, a low positive correlation between PEN-13 and level of education could be shown, as well as a moderate positive correlation between patient enablement and current health status (EQ-VAS). A positive correlation between enablement (PEI score) and level of education has also been found in the study of Groene et al. [55]. A relevant positive correlation between patient enablement, measured by the PEI, and overall health status was also found in two other studies [56,57] but not in a pilot study [58]. Mead et al. even suggest that enablement may be a possible predictor of health-related quality of life, which should be further investigated [59].

In comparison to the PEI [2,17], PEN-13 describes patient enablement in greater detail; at the same time, it does not presuppose any prior intervention but can be used as a general patient-reported outcome across (and independently from) specific medical conditions. PEN-13 is also the first German-language instrument measuring patient enablement. A study published in 2017 [60] indicates that there is an interest in patient enablement assessment. Furthermore, PEN-13 could also be an alternative to PAM-13 for those researchers who consider to assess a patient-related outcome but are not bound and determined to specifically assess patient activation (including the ‘belief component’) or patient empowerment (including the ‘power’ component). As the connotation of the ‘enabled patient’ is smaller than the connotation of both the ‘activated patient’ and ‘empowered patient’, hence its conceptual scope is larger—as has been explained in the introduction (see Figure 1 and Figure 2). Under certain research conditions and perspectives, this could be an advantage.

So far, only one aspect of reliability (internal consistency) has been checked; thus PEN-13′s test-retest reliability remains to be demonstrated. This holds also for the responsiveness of the instrument.

We should be aware of a possible response bias in our study: Participants who responded to our survey are likely to be those who are concerned with health care and personal health and have a greater interest in these issues. Furthermore, it is unclear whether and to what extent the selection of the survey participants as enrollees of a regional integrated health care system limits the external validity of the results. Therefore we consider it reasonable to verify the results in a different sample or setting. Furthermore, the study design did not allow insights into the process, causalities and predictive power of patient enablement. In future studies, the responsiveness of the instrument should be checked.

## 5. Conclusions

The newly developed and validated instrument PEN-13 can be used to assess patient enablement generically, i.e., independently of certain medical conditions, and without presupposing prior interventions. PEN-13 represents an operationalization of patient enablement which helps to objectify the interrelationship with—and possibly the causal influence on—medical outcomes in the future. We recommend further research regarding the test-retest reliability and the responsiveness of the instrument. This would create also a solid evidence base for using PEN-13 in the evaluation of interventions.

### Practical Implications

For the first time, a validated instrument for the comprehensive measurement of patient enablement is provided in German. Future research should be carried out with regard to the responsiveness of the instrument and interpretability of the PEN-13 scores. Also steps should be undertaken to translate the PEN-13 into other languages and its adaptation to other cultural settings and validate these versions in their respective contexts.

## Figures and Tables

**Table 1:** Socio-demographic characteristics of the study participants.

	Total		Calibration Sample		Validation Sample	
	*N* = 1168		*n* = 584		*n* = 584	
	*n*	%	*n*	%	*n*	%
Gender						
Male	506	43.3	255	43.7	251	43.0
Female	662	56.7	329	56.3	333	57.0
Age (Years)						
Mean (Standard Deviation)	62.3 (15.9)		61.65 (15.8)		63.00 (16.0)	
Range	19–95		19–92		19–95	
Chronic Disease						
Yes	650	55.7	327	56.0	323	55.3
No	412	35.3	212	36.3	200	34.2
Don’t know	70	6.0	25	4.3	45	7.7
Missing	36	3.1	20	3.4	16	2.7
Education level						
No school leaving certificate	19	1.6	11	1.9	8	1.4
Secondary school certificate	735	62.9	363	62.2	372	63.7
Intermediate maturity	255	21.8	130	22.3	125	21.4
Polytechnic secondary school	11	0.9	5	0.9	6	1.0
Advanced technical college certificate	58	5.0	30	5.1	28	4.8
Abitur (a-level)	45	3.9	19	3.3	26	4.5
Missing	45	3.9	26	4.5	19	3.3
Employment status						
Currently employed	497	42.6	256	43.8	241	41.3
Currently not employed	587	50.3	292	50.0	295	50.5
Missing	84	7.1	36	6.2	48	8.2

**Table 2:** Description of the scores for health-related quality of life (EQ-5D index), health status (EQ-VAS), health literacy (HLS-EU-Q16) and General Self-Efficacy (GSE).

	Total		Calibration Sample		Validation Sample	
	*n*	Mean (SD)	*n*	Mean (SD)	*n*	Mean (SD)
EQ-5D Index	1090	0.84 (0.20)	544	0.84 (0.20)	546	0.83 (0.20)
EQ-VAS	1052	68.69 (20.30)	527	69.11 (19.92)	525	68.27 (20.69)
HLS-EU-Q16	-		-		126	12.19 (4.18)
GSE scale	-		-		162	29.01 (5.58)

**Table 3:** Item descriptives in the total sample (*N* = 1168).

	Item ^1^	Factor Loading Factor 1	Factor Loading Factor 2	Mean (SD) ^2^	Missing Values n (%) ^3^
1	I know how I can promote my health.	**0.70**	0.16	4.12 (0.86)	51 (4.5)
2	It is easy for me to practice health-promoting behavior in everyday life (e.g., nutrition, exercise).	**0.71**	0.01	3.72 (0.94)	49 (4.2)
3	I am well informed regarding my health condition.	**0.59**	0.36	4.20 (0.87)	73 (6.3)
4	I am able to cope with my health problems.	**0.74**	0.30	4.01 (0.88)	58 (5.0)
5	I know various treatment possibilities for my diseases.	**0.66**	0.24	3.71 (1.0)	113 (9.7)
6	I am able to prevent a deterioration of my health condition as much as this is possible.	**0.75**	0.22	3.83 (0.94)	79 (6.8)
7	I know when to seek medical or therapeutic help, or when I can deal with my complaints on my own.	**0.63**	0.45	3.99 (0.91)	56 (4.8)
8	I am able to get medical or therapeutic help when I need it.	0.50	**0.63**	4.26 (0.88)	50 (4.3)
9	I have no difficulty in telling my doctor about my concerns and fears, even if he or she does not address them directly.	0.18	**0.90**	4.12 (0.98)	37 (3.2)
10	It is easy for me to ask my questions or express my wishes during a medical consultation.	0.19	**0.87**	4.19 (0.95)	35 (3.0)
11	I am convinced that I can practice a healthy lifestyle even in strenuous times.	**0.64**	0.32	3.63 (0.96)	39 (3.3)
12	In general, I am coping well with life.	**0.63**	0.37	4.19 (0.86)	26 (2.2)
13	On the whole, I am able to look after myself.	**0.65**	0.34	4.04 (0.94)	30 (2.6)

^1^ The items presented here reflect a culturally adapted provisional English version of the German PEN-13 version. The initial question to the items was “To what extent do you agree with the following statements for you as a patient?”, and the items were as follows: ^2^ SD—standard deviation; scale 1–5: 1—strongly disagree; 2—disagree; 3—neither/nor; 4—agree; and 5—strongly agree. ^3^ For each study participant with one to three missing items, these were substituted by the mean of the respondent’s valid items. The bold marking shows the assignment to the factor.

**Table 4:** Inter-item correlation matrix of PEN-13 items in the total sample.

	Item_1	Item_2	Item_3	Item_4	Item_5	Item_6	Item_7	Item_8	Item_9	Item_10	Item_11	Item_12	Item_13
Item_1	1	0.506	0.462	0.479	0.521	0.482	0.501	0.429	0.316	0.358	0.426	0.412	0.443
Item_2	0.506	1	0.361	0.395	0.377	0.449	0.397	0.327	0.242	0.274	0.524	0.378	0.372
Item_3	0.462	0.361	1	0.622	0.539	0.512	0.513	0.517	0.438	0.447	0.412	0.368	0.357
Item_4	0.479	0.395	0.622	1	0.552	0.619	0.583	0.54	0.406	0.411	0.494	0.553	0.564
Item_5	0.521	0.377	0.539	0.552	1	0.556	0.523	0.445	0.388	0.367	0.428	0.378	0.423
Item_6	0.482	0.449	0.512	0.619	0.556	1	0.636	0.52	0.375	0.345	0.517	0.492	0.528
Item_7	0.501	0.397	0.513	0.583	0.523	0.636	1	0.66	0.475	0.444	0.458	0.484	0.539
Item_8	0.429	0.327	0.517	0.54	0.445	0.52	0.66	1	0.586	0.55	0.437	0.536	0.562
Item_9	0.316	0.242	0.438	0.406	0.388	0.375	0.475	0.586	1	0.775	0.423	0.423	0.382
Item_10	0.358	0.274	0.447	0.411	0.367	0.345	0.444	0.55	0.775	1	0.457	0.41	0.391
Item_11	0.426	0.524	0.412	0.494	0.428	0.517	0.458	0.437	0.423	0.457	1	0.558	0.508
Item_12	0.412	0.378	0.368	0.553	0.378	0.492	0.484	0.536	0.423	0.41	0.558	1	0.732
Item_13	0.443	0.372	0.357	0.564	0.423	0.528	0.539	0.562	0.382	0.391	0.508	0.732	1

**Table 5:** Corrected item-total correlation of PEN-13 items in the total sample.

Item_1	Item_2	Item_3	Item_4	Item_5	Item_6	Item_7	Item_8	Item_9	Item_10	Item_11	Item_12	Item_13
0.619	0.527	0.645	0.728	0.637	0.704	0.728	0.715	0.606	0.607	0.657	0.666	0.673

**Table 6:** Confirmatory analysis of the two-factor model (without and with adjustment) and the one-factor model.

Criterion	Two-Factor Model Without Adjustment	Two-Factor Model with Two Additional Correlations ^1^	One-Factor Model	One-Factor Model with Two Additional Correlations ^1^
Chi^2^	768.357	443.723	992.045	495.927
*p*-value of Chi^2^	<0.001	<0.001	<0.001	<0.001
Df	64	62	65	63
Chi^2^/df	12.006	7.157	15.262	7.872
Comparative Fit Index (CFI)	0.821	0.903	0.765	0.890
Tucker Lewis Index (TLI)	0.782	0.878	0.718	0.864
Root Mean Square Error of Approximation (RMSEA) (90% CI)	0.137 (0.129–0.146)	0.103 (0.094–0.112)	0.156 (0.148–0.165)	0.109 (0.100–0.118)

^1^ Intercorrelation between the error terms of the items 12 and 13 (factor 1) and 9 and 10 (factor 2).

## Supplementary Materials

The following are available online at https://www.mdpi.com/1660-4601/16/23/4867/s1, Table S1: Factor loading after Varimax rotation in the calibration sample.

## Availability of the German version of PEN-13

The German version of the PEN-13 can be requested from our work group—please contact the first author.
