# Correction: Siegel et al. PEN-13: A New Generic 13-Item Questionnaire for Measuring Patient Enablement (German Version). *Int. J. Environ. Res. Public Health* 2019, *16*, 4867

**DOI:** 10.3390/ijerph21040411

**Published:** 2024-03-28

**Authors:** Achim Siegel, Anna T. Ehmann, Ingo Meyer, Oliver Gröne, Wilhelm Niebling, Peter Martus, Monika A. Rieger

**Affiliations:** 1 Institute of Occupational and Social Medicine and Health Services Research, University Hospital Tübingen, Wilhelmstraße 27, 72074 Tübingen, Germany; anna.ehmann@med.uni-tuebingen.de (A.T.E.); monika.rieger@med.uni-tuebingen.de (M.A.R.); 2 PMV Forschungsgruppe, University of Cologne, Herderstraße 52, 50391 Cologne, Germany; ingo.meyer@uk-koeln.de; 3 OptiMedis AG, Burchardstraße 17, 20095 Hamburg, Germany; o.groene@optimedis.de; 4 London School of Hygiene and Tropical Medicine, University of London, London WC1E 7HT, UK; 5 Division of General Practice, University Medical Center Freiburg, 79910 Freiburg, Germany; wilhelm.niebling@googlemail.com; 6 Institute for Clinical Epidemiology and Applied Biometry, University Hospital Tübingen, Silcherstr. 5, 72076 Tübingen, Germany; peter.martus@med.uni-tuebingen.de

In the original publication [1], the wording of the provisional English translation of questionnaire items 5, 7, and 11 in Table 3 contained some linguistic inaccuracies; the wording of these three items has now been corrected. The corrected Table 3 appears below. The authors state that the scientific conclusions are unaffected.

## Figures and Tables

**Table 3:** Item descriptives in the total sample (*N* = 1168).

	Item ^1^	Factor Loading Factor 1	Factor Loading Factor 2	Mean (SD) ^2^	Missing Values n (%) ^3^
1	I know how I can promote my health.	**0.70**	0.16	4.12 (0.86)	51 (4.5)
2	It is easy for me to practice health-promoting behavior in everyday life (e.g., nutrition, exercise).	**0.71**	0.01	3.72 (0.94)	49 (4.2)
3	I am well informed regarding my health condition.	**0.59**	0.36	4.20 (0.87)	73 (6.3)
4	I am able to cope with my health problems.	**0.74**	0.30	4.01 (0.88)	58 (5.0)
5	I know various treatment possibilities for my diseases.	**0.66**	0.24	3.71 (1.0)	113 (9.7)
6	I am able to prevent a deterioration of my health condition as much as this is possible.	**0.75**	0.22	3.83 (0.94)	79 (6.8)
7	I know when to seek medical or therapeutic help, or when I can deal with my complaints on my own.	**0.63**	0.45	3.99 (0.91)	56 (4.8)
8	I am able to get medical or therapeutic help when I need it.	0.50	**0.63**	4.26 (0.88)	50 (4.3)
9	I have no difficulty in telling my doctor about my concerns and fears, even if he or she does not address them directly.	0.18	**0.90**	4.12 (0.98)	37 (3.2)
10	It is easy for me to ask my questions or express my wishes during a medical consultation.	0.19	**0.87**	4.19 (0.95)	35 (3.0)
11	I am convinced that I can practice a healthy lifestyle even in strenuous times.	**0.64**	0.32	3.63 (0.96)	39 (3.3)
12	In general, I am coping well with life.	**0.63**	0.37	4.19 (0.86)	26 (2.2)
13	On the whole, I am able to look after myself.	**0.65**	0.34	4.04 (0.94)	30 (2.6)

^1^ The items presented here reflect a culturally adapted provisional English version of the German PEN-13 version. The initial question to the items was “To what extent do you agree with the following statements for you as a patient?”, and the items were as follows: ^2^ SD—standard deviation; scale 1–5: 1—strongly disagree; 2—disagree; 3—neither/nor; 4—agree; and 5—strongly agree. ^3^ For each study participant with one to three missing items, these were substituted by the mean of the respondent’s valid items. The bold marking shows the assignment to the factor.

## References

[1] Siegel A., Ehmann A.T., Meyer I., Gröne O., Niebling W., Martus P., Rieger M.A. (2019). PEN-13: A New Generic 13-Item Questionnaire for Measuring Patient Enablement (German Version). Int. J. Environ. Res. Public Health.

